# The percentage and clinical correlates of alexithymia in stable patients with schizophrenia

**DOI:** 10.1007/s00406-022-01492-8

**Published:** 2022-10-14

**Authors:** Yun Yi, Yuanyuan Huang, Rui Jiang, Qiang Chen, Mingzhe Yang, Hehua Li, Yangdong Feng, Shixuan Feng, Sumiao Zhou, Lixin Zhang, Yuping Ning, Zezhi Li, Fengchun Wu

**Affiliations:** 1 Department of Psychiatry, The Affiliated Brain Hospital of Guangzhou Medical University, 36 Mingxin Rd, Liwan District, Guangzhou, 510370 China; 2 Department of Psychiatry, Qingdao Mental Health Center, Qingdao, China; 3 Department of Psychiatry, The Brain Hospital of Guangxi Zhuang Autonomous Region, Guangxi, China; 4 Guangdong Engineering Technology Research Center for Translational Medicine of Mental Disorders, Guangzhou, China

**Keywords:** Alexithymia, Schizophrenia, Negative symptoms, Depressive symptoms

## Abstract

Alexithymia is a common, but less-recognized affective deficit in patients with schizophrenia. To date, no definitive conclusions have been drawn about the relationship between alexithymia and the clinical symptoms or their clinical correlates, particularly in stable patients with schizophrenia. The purpose of this study was to investigate the link between alexithymia and psychopathological symptoms, as well as any associated correlates, in stable patients with schizophrenia. A total of 435 Chinese patients with schizophrenia were recruited. The Positive and Negative Symptoms Scale (PANSS) was used to evaluate each patient’s psychopathological symptoms. The Toronto Alexithymia Scale (TAS-20) was used to measure alexithymia. The percentage of alexithymia was 35.2% in stable patients with schizophrenia. Compared to non-alexithymia patients, patients with alexithymia had higher PANSS total scores, negative subscores, depressive subscores, and cognitive subscores (all *p* < 0.05). Multivariate regression analysis revealed that the following variables were positively associated with TAS-20 total scores: PANSS negative subscores (*β* = 0.274, *t* = 3.198, *p* = 0.001) and PANSS depressive subscores (*β* = 0.366, *t* = 2.500, *p* = 0.013). Education years (*β* = – 0.453, *t* = – 2.824, *p* = 0.005) was negatively associated with TAS-20 total scores. Our results suggest that the percentage of alexithymia was relatively higher in stable patients with schizophrenia. Education levels, negative symptoms, and depressive symptoms were independently associated with alexithymia in this specific population.

## Introduction

Previous studies have largely focused on the cognitive function and demonstrate that neurocognitive impairment is a core feature of schizophrenia [1]. In recent years, social cognition has gradually been paid more attention to. Social cognition is an umbrella theoretical construct that refers to the ability for individuals to perceive, interpret, and process social information for interactions [2, 3]. It includes emotional perception, theory of mind, social perception, and attributional style [3]. Alexithymia belongs to another important aspect of social cognitive and has not been investigated to a great extent. The term alexithymia was derived from Greek (a = lack, lexis = word, thymos = emotion) to describe deficiencies in emotional functioning [4, 5]. Alexithymia, which literally means “absence of words for feelings,” is characterized by cognitive–affective disturbances and involves impairments in identifying and expressing one’s feelings verbally [6].

Patients with schizophrenia experience difficulties in dealing with emotions, which is evidenced by having lowered or disturbed emotional perception, emotional experience, emotional expression, and emotional identification [7]. Previous studies had shown that alexithymia can exist in both acute and stable stages of schizophrenia [8]. Relatively limited data have suggested that the prevalence of alexithymia in schizophrenia populations ranges from 30 to 46% [9, 10], which is significantly higher than in the general population [11]. Patients with schizophrenia with lack of emotional awareness associated with alexithymia have been demonstrated to influence personal satisfaction [12] and social functioning [13], as well as to impair one’s ability to interact with people and form close, meaningful connections [14]. Alexithymia is not regarded as a disorder in the Diagnostic and Statistical Manual. Alexithymia is regularly assessed using the self-report scale known as Toronto Alexithymia Scale (TAS) rather than the diagnostic criteria [15]. Therefore, the study about alexithymia in patients with schizophrenia should focus on the stable episode rather than acute episode, which was due to the characteristics of disease.

Several studies have explored the relationship between alexithymia and demographic factors and clinical psychopathology, but the results have been inconsistent. Wout et al. showed that male patients with schizophrenia have particular alexithymia patterns which make it difficult to recognize and express emotions [16]. Kubota et al. recruited 21 patients with schizophrenia and revealed that both men and women faced obstacles identifying and describing their own emotions [17]. They also showed that there was no association between alexithymia and illness duration or antipsychotics. With regards to clinical psychopathology, previous reports have revealed that patients with schizophrenia without social drive and curbing of interests demonstrate strong correlations between alexithymia and psychopathological symptoms, particularly negative psychopathological symptoms [18]. Furthermore, Fogley et al. demonstrated that higher levels of alexithymia are associated with greater emotional discomfort [19]. Some studies, however, have questioned links between alexithymia and psychotic symptoms and showed that alexithymia is not related to the severity of psychopathological symptoms (i.e., positive or negative symptom dimensions) [8, 17, 19, 20]. Todarello et al. reported that the severity of alexithymia remained stable even when psychiatric symptoms showed improvement throughout a year of treatment, indicating that alexithymia was an independent construct from schizophrenia [8]. The inconsistencies of these results may be due to the heterogeneity of schizophrenia individuals, which were caused by different comorbidities of disorder such as anxiety, depression or personality disorder. Fogley et al. pointed that anxiety and depression were associated with alexithymia. Furthermore, anxiety and depression may impair the ability or motivation to express emotions [19]. Yu et al. revealed the relationship between personality disorder and alexithymia in patients with schizophrenia [21]. For example, they found that the antisocial personality disorder can predict difficulty identifying feelings due to lacking of empathy and showing indifference to others. The avoidant style predicted difficulty describing feelings because of the increasing of introversion and neuroticism. The histrionic and paranoid personality disorder can predict externally oriented thinking because of focusing on concrete details of external and high vigilance. Therefore, the differences of comorbidities contribute to the alexithymia in patients with schizophrenia.

To date, no definitive conclusions have been drawn about the relationship between alexithymia and clinical symptoms or the clinical correlates of that relationship, and the relevant studies in stable patients with schizophrenia are very few. The aim of this study, therefore, was to investigate: (1) the percentage of alexithymia in stable patients with schizophrenia; (2) the association between alexithymia and clinical psychopathological symptoms in stable patients with schizophrenia; and (3) any demographic factors correlated of alexithymia.

## Subjects and methods

### Subjects

This study belonged to a cross-section design and was approved by the Institutional Review Board (IRB) of the Affiliated Brain Hospital of Guangzhou Medical University. All patients were recruited from this institute including outpatients and inpatients between March 2018 and September 2019. All participating patients provided written informed consent. Inclusion criteria were as follows: 1) satisfied the DSM-IV criteria for schizophrenia; 2) aged 18–50 years; 3) ≥ 1 year illness durations; 4) had been on stable antipsychotics at least 6 months; 5) a member of the Han Chinese population. The criteria of exclusion were as follows: 1) experiencing pregnancy or lactation; 2) diagnosis of any other major Axis I disorder; 3) comorbid physical, infectious, immune system illnesses, or mental retardation; 4) drug or alcohol dependence; 5) psychotic symptoms fluctuating within 2 weeks.

### Clinical interview and measurements

The Structured Clinical Interview for DSM-IV Axis I Disorders-Patient Edition (SCID-I/P) was used by two trained psychiatrists to screen the participants. A self-designed questionnaire was used to collect basic information, social-demographic factors, and medical conditions. We also collected additional information from medical records.

We conducted the Positive and Negative Syndrome Scale (PANSS) to evaluate schizophrenia symptoms [22]. The five-factor PANSS model was adopted to assess patients’ psychopathology [23, 24]: PANSS positive factors were determined by P1, P3, P5, P6, and G9; PANSS negative factors were determined by N1, N2, N3, N4, N6, G7, and G16; PANSS depressive factors were determined by G1, G2, G3, G4, and G6; PANSS cognitive factors were determined by P2, N5, N7, G5, G10, G11, G12, G13, and G15; PANSS excited factors were determined by P4, P7, G8, and G14. All research workers attended a training session related to the PANSS. Further, the inter-observer correlation coefficient (ICC) was above the stated critical point of 0.8 [25].

### Alexithymia assessment

The Toronto Alexithymia Scale (TAS-20) was used to measure sensation identification and descriptions [26], with higher scores on the different subscales indicating poorer functioning. This is a commonly used and multidimensional self-report instrument that makes use of a three-factor structure: difficulty in identifying feelings (DIF), difficulty in describing feeling (DDF), and externally oriented thinking (EOT). Each item was evaluated using a 5-point Likert scale, and the answers within each relevant item were added together to form a total subscale score. The total alexithymia score was generated from the sum of all responses. In order to determine alexithymia percentage, the total TAS-20 score was classified based on the critical point proposed by Bagby [26]. Based on this classification scheme, a score of ≤ 51 indicates no alexithymia (low), scores between 52–60 indicates possible alexithymia (medium), and scores of ≥ 61 indicate alexithymia (high). The Chinese version was translated by Zhu [27], and the TAS-20 (Chinese version) demonstrates high internal coherence (Cronbach's alpha = 0.83), reliability test-record (0.87), and convergent validity [28].

### Statistical analysis

All the continuous data were examined using Kolmogorov–Smirnov one-sample tests to verify normality. Demographic and clinical data across the three groups were compared using one-way ANOVAs for continuous variables and chi-square tests for classified variables. The correlation coefficient matrix between clinical symptoms and alexithymia was calculated using Pearson correlation analyses on each PANSS score and the matching TAS-20 total scores and subscales. The false discovery rate (FDR) correction was used to adjust for multiple tests. We then performed partial correlations by controlling covariates (including sex, age, BMI, education, marital status, smoke status, family history of psychotic disease, age of onset, times of hospitalizations, antipsychotic drug use (converted to an equivalent chlorpromazine dose [29])) and investigating the interconnections between clinical symptoms and alexithymia. Multiple linear stepwise regression analysis was applied to identify which characteristics were most substantially associated with total alexithymia scores or with any of its three subscales. We diagnosed multicollinearity by referring to the values provided by the Tolerance and Variance expansion factors (VIF), because a strong correlation between predictor variables may have resulted in multicollinearity in the prediction (tolerance values less than 0.10 and VIF values more than 10 indicate the presence of multicollinearity). All statistical analyses were carried out using SPSS version 25.0, and statistical significance was determined as a 2-tailed p value that was less than 0.05.

## Results

### Demographic and clinical characteristics of patients

**Fig. 1:**
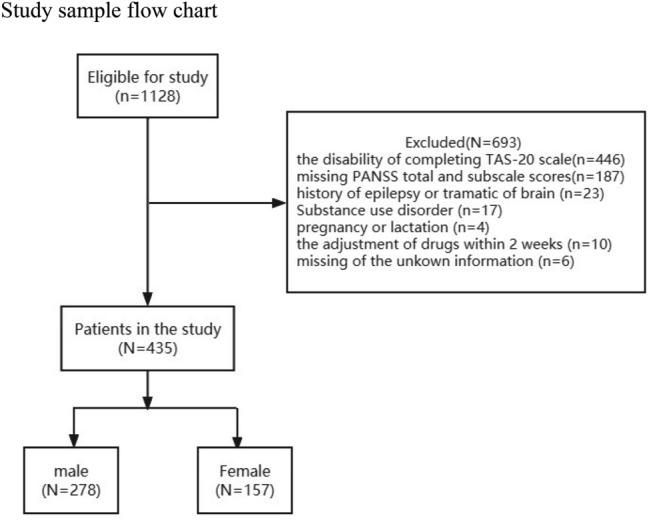
Study sample flowchart

A total of 1128 patients were screened, with 435 patients (male 63.9%, female 36.1%) eventually included in the study. Table 1 shows the sociodemographic characteristics of the total sample. A detailed sample flowchart is shown in Fig. 1.

**Table 1:** Characteristics of the sample

	*N* (%)	*M* (SD)
Age	435	37.9 (8.1)
Education (years)	431	9.4 (3.3)
BMI	435	25.1 (4.7)
Onset age	435	23.4 (6.1)
Numbers of hospitalization	421	7.2 (9.3)
Antipsychotic dose (mg/day)	425	387.5 (843.1)
Gender		
Male	278 (63.9%)	
Female	157 (36.1%)	
Marital status		
Unmarried	279 (64.3%)	
Married	85 (19.6%)	
Divorce	67 (15.4%)	
Widowhood	3 (0.7%)	
Smoking status		
No smoking	255 (60%)	
Ever smoking	72 (16.9%)	
Now smoking	98 (23.1%)	
Family history of psychotic disorder		
Positive	82 (18.9%)	
Negative	353 (81.1%)	

*M* mean, *SD* standard deviation, *BMI* body mass index

Our study showed that the percentage of alexithymia in patients with schizophrenia was 35.2%. As shown in Table 2, patients were divided into three groups according to the severity of alexithymia. The number of individuals with low TAS-20 (≤ 51) scores in this study was 116 (26.7%), the number of individuals with medium TAS-20 (52 ~ 60) scores was 166 (38.1%), and the number of individuals with high TAS-20 (≥ 61) scores was 153 (35.2%). There were no significant differences among the three alexithymia groups in terms of age, gender, smoking status, marital status, family history status (*p* > 0.05). There were also no significant between-group differences in onset age, the number of hospitalizations, or antipsychotic drug dosage (CPZ equivalent) (*p* > 0.05). However, post-hoc Bonferroni post-analysis following one-way ANOVAs revealed marginally significant variations in education levels (*p*’s < 0.05) and BMI across the three groups. The alexithymia patients had higher PANSS total subscores, negative subscores, cognitive subscores, and depressive subscores compared to non-alexithymia ( all *p* < 0.05) patients, even after Bonferroni corrections (*p* Bonferroni corrected < 0.05).

**Table 2:** Comparison of demographic characteristics and clinical symptoms

Characteristics	Low alexithymia	Moderate alexithymia	High alexithymia	*F*/*χ*^2^	*P*-value
	*N* = 116	*N* = 166	*N* = 153		
Age (years)	38.0 ± 7.9	37.0 ± 8.6	38.4 ± 7.7	0.673	0.511
Education level (years)	10.5 ± 3.8	9.2 ± 2.9	8.8 ± 3.1	8.813	< 0.01*
BMI	24.3 ± 4.8	25.7 ± 5.1	25.2 ± 4.1	3.071	0.047*
Onset age (years)	23.4 ± 6.0	23.3 ± 6.1	23.4 ± 6.2	0.012	0.988
Number of hospitalizations	5.42 ± 6.52	7.60 ± 9.17	8.01 ± 10.89	2.803	0.062
Antipsychotic drug dosage (CPZ equivalent mg)	429.3 ± 940.2	313.3 ± 415.1	444.6 ± 1090.8	1.076	0.342
Male (%)	67 (24.1)	105 (37.8)	106 (38.1)	3.848	0.146
Family history of the psychotic disorder (%)					
Negative	89 (25.2)	13 (37.4)	132 (37.4)	4.401	0.111
Positive	27 (32.9)	34 (41.5)	21 (25.6)		
Status of smoking					
Non-smoking	71 (27.8)	100 (39.2)	84 (32.9)	1.435	0.838
Ever smoking	19 (26.4)	25 (34.7)	28 (38.9)		
Current smoking	24 (24.5)	37 (37.8)	37 (37.8)		
Status marriage					
Non-married	78 (28)	98 (35.1)	103 (36.9)	7.597	0.225
Married	21 (24.7)	42 (49.4)	22 (25.9)		
Divorced	17 (25.4)	24 (35.8)	26 (38.8)		
Widowed	0	2 (66.7)	1 (33.3)		
PANSS					
PANSS total	71.88 ± 16.63	75.73 ± 16.96	78.88 ± 17.92	7.211	0.001**
PANSS positive	12.02 ± 5.43	11.57 ± 4.82	11.76 ± 4.46	0.285	0.752
PANSS negative	17.61 ± 6.54	19.15 ± 6.94	21.30 ± 7.37	9.506	< 0.001**
PANSS cognitive	23.21 ± 6.70	24.72 ± 6.54	26.48 ± 7.91	7.175	0.001**
PANSS depressant	11.27 ± 3.48	12.39 ± 3.64	12.45 ± 3.45	4.548	0.018*
PANSS excited	7.78 ± 3.06	7.90 ± 2.94	7.89 ± 3.22	0.067	0.936

Low = total alexithymia score ≤ 51, moderate = total alexithymia score:52 ~ 60, high = total alexithymia score ≥ 61

*BMI* body mass index, *PANSS* Positive and Negative Syndrome Scale

^*^Indicates that there was a significant difference between alexithymia and demographic factors and clinical symptoms. *p < 0.05, **p < 0.005

### Correlation between clinical symptoms and TAS-20

**Fig.2:**
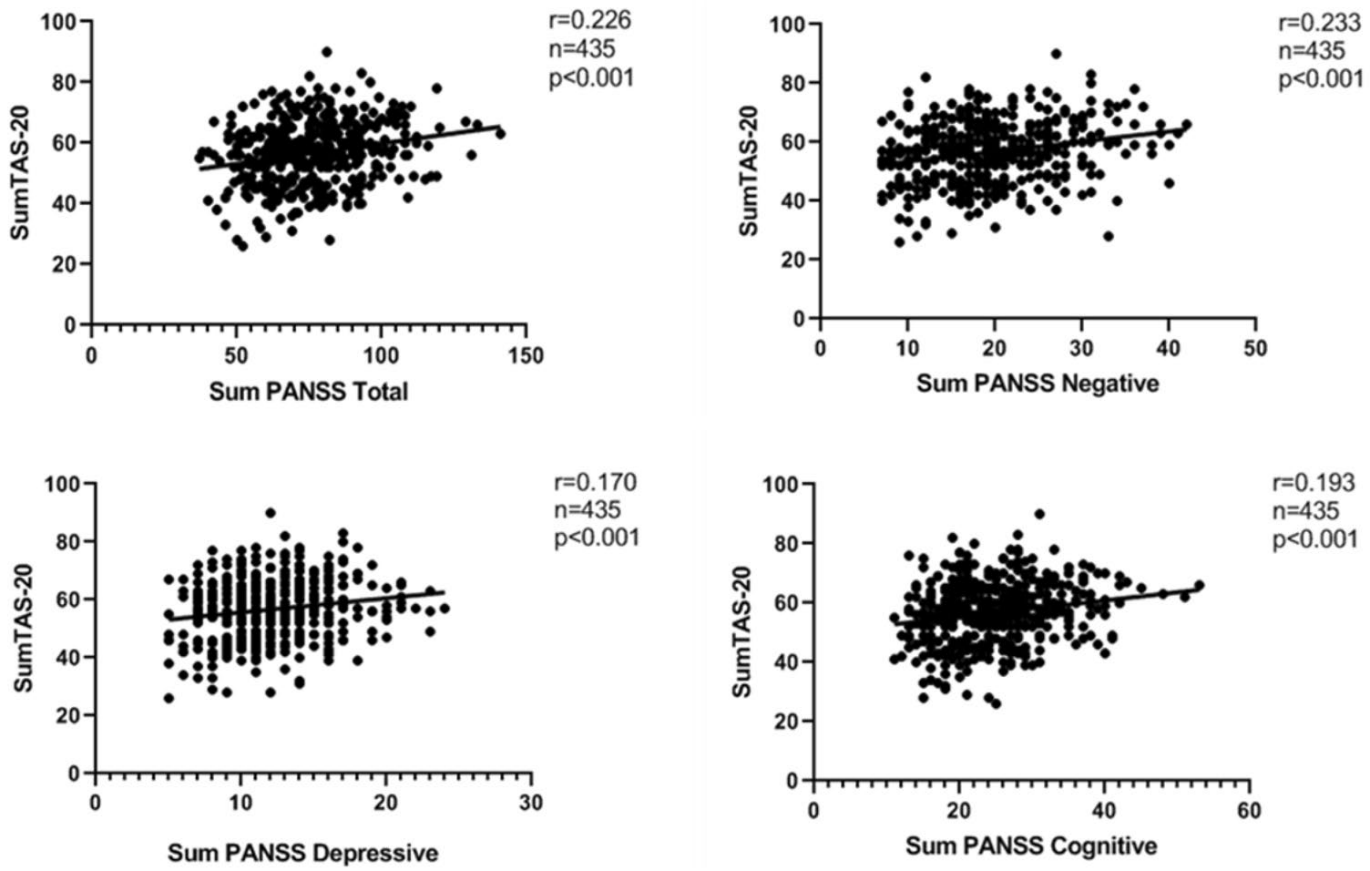
Correlation analysis revealed TAS-20 total scores were positively associated with PANSS total scores, negative symptoms, cognitive symptoms and depressive symptoms

Using Pearson correlation analysis, we created correlation matrices for the relationships between TAS-20 subscales and clinical symptoms. TAS-20 total scores were positively correlated with PANSS total subscores (*r* = 0.226, *p* < 0.01), together with subscales including negative subscores (*r* = 0.233, *p* < 0.001), cognitive subscores (*r* = 0.193, *p* < 0.01) and depressive subscores (*r* = 0.170, *p* < 0.01) (Fig. 2) (all *p*_FDR-correction_ < 0.001). DIF was positively associated with PANSS total subscores (*r* = 0.168, *p* < 0.001), negative subscores (*r* = 0.139, *p* = 0.004), cognitive subscores (*r* = 0.159, *p* < 0.001) and depressive subscores (*r* = 0.130, *p* = 0.007) (all *p*_FDR-correction_ < 0.01). DDF was positively associated with PANSS total subscores (*r* = 0.199, *p* < 0.001), negative subscores (*r* = 0.254, *p* < 0.001), cognitive subscores (*r* = 0.131, *p* = 0.006) and depressive subscores (*r* = 0.156, *p* = 0.001) (all *p*_FDR-correction_ < 0.01). EOT was positively associated with the PANSS total score (*r* = 0.137, *p* = 0.004), negative subscores (*r* = 0.147, *p* = 0.002) and cognitive subscores (*r* = 0.128, *p* = 0.008) (all *p*_FDR-correction_ < 0.01).

We also generated a matrix of partial correlation coefficients through partial correlation. As shown in Table 3, TAS-20 total subscores were positively associated with the PANSS total score (*r* = 0.189, *p* < 0.001), negative subscores (*r* = 0.228, *p* < 0.001), cognitive subscores (*r* = 0.138, *p* = 0.005), and depressive subscores (*r* = 0.159, *p* = 0.001). DIF was positively associated with the PANSS total score (*r* = 0.142, *p* = 0.004), negative subscores (*r* = 0.134, *p* = 0.007), cognitive subscores (*r* = 0.123, *p* = 0.012), and depressive subscores (*r* = 0.129, *p* = 0.009). DDF was positively associated with PANSS total subscores (*r* = 0.181, *p* < 0.001), negative subscores (*r* = 0.263, *p* < 0.001) and depressive subscores (*r* = 0.147, *p* = 0.003). EOT was positively associated with PANSS negative subscores (*r* = 0.124, *p* = 0.011).

**Table 3:** Matrix of partial correlation coefficients between alexithymia and clinical symptoms

	TAS-20	DIF	DDF	EOT
PANSS total	0.189^**^	0.142^**^	0.181^**^	0.091
PANSS negative	0.228^**^	0.134^*^	0.263^**^	0.124^*^
PANSS cognitive	0.138^**^	0.123^*^	0.095	0.073
PANSS depressive	0.159^**^	0.129^*^	0.147^**^	0.070

TAS-20 = the 20-item of Toronto Alexithymia Scale *PANSS* = Positive and Negative Syndrome Scale

*DIF* difficulty identifying feelings. *DDF* difficulty describing feelings. *EOT* externally orientated thinking

^*^Indicates that there was a significant correlation between alexithymia and clinical symptoms. *p < 0.05; **p < 0.005

### Associated factors for alexithymia in schizophrenia patients

Multivariate regression analysis revealed that the following variables were positively associated with TAS-20 total scores: PANSS negative subscores (*β* = 0.274, *t* = 3.198, *p* = 0.001) and PANSS depressive subscores (*β* = 0.366, *t* = 2.500, *p* = 0.013). Education years (*β* = – 0.453, *t* = – 2.824, *p* = 0.005) was negatively associated with TAS-20 total scores, with adjust *R*^2^ = 0.103. DIF was positively associated with the PANSS depressive subscores (*β* = 0.178, *t* = 2.058, *p* = 0.04), with adjust *R*2 = 0.054. DDF was positively associated with the PANSS negative subscores (*β* = 0.153, *t* = 4.891, *p* < 0.001) and depressive subscores (*β* = 0.113, *t* = 2.119, *p* = 0.035). DDF was negatively associated with education (*β* = – 0.134, *t* = – 2.298, *p* = 0.022), with adjusted R^2^ = 0.088. EOT factors were negatively associated with education years (*β* = – 0.174, *t* = – 2.444, *p* = 0.015), with adjusted *R*^2^ = 0.040. Tolerance and VIF values did not show any multicollinearity (all tolerance > 0.1 or VIF < 10).

## Discussion

The primary results of this study included: (1) alexithymia percentage in patients with schizophrenia was 35.2%; (2) PANSS negative symptoms and depressive symptoms were positively associated with alexithymia; (3) education levels were inversely associated with alexithymia.

Our study found an alexithymia percentage of 35.2%, which was nearly consistent with a previous study (30% percentage in outpatients) [9]. Additionally, alexithymia percentage in our study cohort was much higher than that previously observed (i.e., an 5–14% percentage in the general population) [30]. Patients with schizophrenia had been hospitalized for extended periods, and these closed hospitalization environments restricted their ability to communicate with family and friends. Another plausible explanation is that chronic patients with schizophrenia could also be suffering from the symptoms of the diseases themselves, including social withdrawal, emotional indifference, and decreased initiative, all of which could have increased the percentage of alexithymia. However, McGillivray et al. defined 64 on the TAS-20 as the cutoff for alexithymia and calculated a 45.7% prevalence [10]. Thus, these differences could be attributed to differing definitions of alexithymia. Our results indicated that patients with schizophrenia in stable stages also had a relatively high alexithymia level.

We found a positive relationship between alexithymia (mainly based on total TAS-20 scores and DDF) and negative PANSS symptoms, which was consistent with previous findings [18, 31]. Possible explanations include, first, theoretically, negative schizophrenia symptoms are compatible with alexithymia, and the same symptomatology is responsible for the link between alexithymia and negative symptoms. Second, Rahm et al. showed that changes in amygdala structure and function were associated with negative symptoms and decreased emotional expression in schizophrenia [32]. It also appears that dysfunction in these brain areas could be the neurological foundation for alexithymia [33, 34]. Thus, another possibility is that alexithymia and negative symptoms of schizophrenia may be linked by the same neurological substrates. A third potential cause may be related to personality traits, which influence individuals’ social behaviors. Negative symptoms have been shown to be substantially related to the harm-avoidance personality dimension [35]. People exhibiting avoidance traits find it difficult to communicate and hide their feelings. Thus, their capacity to share their feelings with others was hampered by their self-imposed social isolation [36]. However, previous research has also produced conflicting results. Gaweda et al. performed a study on 60 patients with schizophrenia and showed that the severity of hallucination was related to alexithymia [20]. Todarello et al. recruited 29 outpatients with schizophrenia and showed that alexithymia was not associated with negative symptoms [8]. The discrepancies in results across those studies may be due to methodological differences, including different sample sizes [8], differing assessment tests, changes in research design, and/or differences in clinical characteristics (e.g., severity and illness duration, age of disease onset, and the number of episodes).

We also found that depressive PANSS symptoms were positively correlated with the TAS-20 total scale, DIF scale, and DDF scale. This indicates that the severity of depression can increase the occurrence of alexithymia, which has also been found in some previous research [37–40]. One possible explanation for this phenomenon is that depression can weaken the capability or motivation to express emotions. Patients with depression regularly applied emotional suppression strategies to deal with symptoms, so it was more difficult for them to describe their emotions freely [40]. Additionally, secondary alexithymia could occur as a result of the depressed symptoms themselves. According to the theory of secondary alexithymia, alexithymia may be a defense strategy to deal with emotional pain, which would mean that people with negative emotions may have high alexithymia levels [41]. In the end, depressive symptoms have the potential to cause social isolation, which creates an atmosphere that is unsuitable for emotional expression. However, some other studies did not report an association between depressive symptoms and alexithymia. Yu et al. recruited 60 paranoid patients with schizophrenia and showed that depressive symptoms were not associated with alexithymia [21]. Van der et al. conducted a study including four different groups and showed a relationship between alexithymia and depressive symptoms in siblings of schizophrenia patients and controls, but not within the schizophrenia group [42]. Taken together, this evidence suggests that there may be an internal relationship between depressive symptoms and alexithymia, but further study is needed in larger-scale samples to increase the understanding of the relationship between depressive symptoms and alexithymia.

We also found that the education levels were negatively associated with the TAS-20 total scale (which captures dimensions related to difficulties describing feelings and external oriented thinking), in line with previous studies [43, 44]. The relationship means the lower the level of education, the greater the alexithymia. When comparing less well-educated patients to highly educated ones, fewer educated individuals had problems detecting and comprehending others’ emotions. At the same time, patients with lower education levels also had lower levels of psychological awareness and emotional intelligence, which could lead to increased difficulties in articulating and coping with their feelings, and could have made them more susceptible to alexithymia. Additionally, having lower levels of education may be associated with poor communication skills and inadequate social contact, both of which may contribute to the incidence of alexithymia in the population.

There are some strengths in the study that should be highlighted: first, alexithymia is valued by self-report scale, which makes it difficult for acute patients with schizophrenia to accomplish the assessment. Therefore, since alexithymia is an inner state [45], our study recruited the patients in stable episode, which made our results more authenticity and validity. Second, this was the first study recruiting large sample of stable patients with schizophrenia in China to investigate the related factors of alexithymia.

There were several limitations to our study. First, included patients had been treated with various antipsychotics. Although these antipsychotics were converted to equivalent chlorpromazine doses, all possible confounding effects might not have been eliminated. A future study recruiting never-treated first-episode schizophrenia patients should be conducted to validate our results in this specific population. Second, we used the TAS-20 scale to measure the severity of alexithymia, but this scale is a self-report evaluation instrument. In the future, a third-party scale could be used to evaluate alexithymia in schizophrenia patients. Third, this cross-sectional study cannot draw definitive conclusions about causal relationships between clinical correlates and alexithymia. Forth, since we did not recruit healthy controls for comparison, whether the percentage of alexithymia in patients with schizophrenia was higher than that in healthy controls should be interpreted carefully. Finally, although we discovered correlations between alexithymia and clinical symptoms of schizophrenia, the potential mechanisms underlying the alexithymia in schizophrenia remained largely unknown.

 In conclusion, this study showed that the percentage of alexithymia was relatively higher in stable patients with schizophrenia and that education levels, negative symptoms, and depressive symptoms were independently associated with alexithymia in this specific population. Clinicians should increase the screening of alexithymia in patients with schizophrenia. Our results can improve our understanding of alexithymia, which can help us make diagnosis of it at an early stage. Thus, we can develop more effective intervention strategies to reduce the negative impact induced by alexithymia. Further it is recommended to train patients with alexithymia and introduce psychological intervention and cognitive training to encourage them to identify and describe their emotions and feelings in an appropriate way. At last, patients with schizophrenia can establish and maintain meaningful and close relationships with others.

## References

[1] Bora E, Binnur Akdede B, Alptekin K (2017). Neurocognitive impairment in deficit and non-deficit schizophrenia: a meta-analysis. Psychol Med.

[2] Pinkham AE, Penn DL, Perkins DO, Lieberman J (2003). Implications for the neural basis of social cognition for the study of schizophrenia. Am J Psychiatry.

[3] Green MF, Olivier B, Crawley JN, Penn DL, Silverstein S (2005). Social cognition in schizophrenia: recommendations from the measurement and treatment research to improve cognition in schizophrenia new approaches conference. Schizophr Bull.

[4] Sifneos PE (1973). The prevalence of 'alexithymic' characteristics in psychosomatic patients. Psychother Psychosom.

[5] Sifneos PE, Apfel-Savitz R, Frankel FH (1977). The phenomenon of 'alexithymia'. Observations in neurotic and psychosomatic patients. Psychother Psychosom.

[6] Taylor GJ, Bagby RM, Parker JD (1991). The alexithymia construct A potential paradigm for psychosomatic medicine. Psychosomatics.

[7] Trémeau F (2006). A review of emotion deficits in schizophrenia. Dialog Clin Neurosci.

[8] Todarello O, Porcelli P, Grilletti F, Bellomo A (2005). Is alexithymia related to negative symptoms of schizophrenia? A preliminary longitudinal study. Psychopathology.

[9] Cedro A, Kokoszka A, Popiel A, Narkiewicz-Jodko W (2001). Alexithymia in schizophrenia: an exploratory study. Psychol Rep.

[10] McGillivray L, Becerra R, Harms C (2017). Prevalence and demographic correlates of alexithymia: a comparison between Australian psychiatric and community samples. J Clin Psychol.

[11] Fukunishi I, Berger D, Wogan J, Kuboki T (1999). Alexithymic traits as predictors of difficulties with adjustment in an outpatient cohort of expatriates in Tokyo. Psychol Rep.

[12] Mattila AK, Poutanen O, Koivisto AM, Salokangas RK, Joukamaa M (2007). Alexithymia and life satisfaction in primary healthcare patients. Psychosomatics.

[13] van Rijn S, Schothorst P, Wout M, Sprong M, Ziermans T, van Engeland H (2011). Affective dysfunctions in adolescents at risk for psychosis: emotion awareness and social functioning. Psychiatry Res.

[14] Ospina LH, Shanahan M, Perez-Rodriguez MM, Chan CC, Clari R, Burdick KE (2019). Alexithymia predicts poorer social and everyday functioning in schizophrenia and bipolar disorder. Psychiatry Res.

[15] Gaggero G, Bonassi A, Dellantonio S, Pastore L, Aryadoust V, Esposito G (2020). A scientometric review of alexithymia: mapping thematic and disciplinary shifts in half a century of research. Front Psych.

[16] van 't Wout M, Aleman A, Bermond B, Kahn RS,  (2007). No words for feelings: alexithymia in schizophrenia patients and first-degree relatives. Compr Psychiatry.

[17] Kubota M, Miyata J, Hirao K, Fujiwara H, Kawada R, Fujimoto S (2011). Alexithymia and regional gray matter alterations in schizophrenia. Neurosci Res.

[18] Tang XW, Yu M, Duan WW, Zhang XR, Sha WW, Wang X (2016). Facial emotion recognition and alexithymia in Chinese male patients with deficit schizophrenia. Psychiatry Res.

[19] Fogley R, Warman D, Lysaker PH (2014). Alexithymia in schizophrenia: associations with neurocognition and emotional distress. Psychiatry Res.

[20] Gawęda Ł, Krężołek M (2019). Cognitive mechanisms of alexithymia in schizophrenia: Investigating the role of basic neurocognitive functioning and cognitive biases. Psychiatry Res.

[21] Yu S, Li H, Liu W, Zheng L, Ma Y, Chen Q (2011). Alexithymia and personality disorder functioning styles in paranoid schizophrenia. Psychopathology.

[22] Kay SR, Fiszbein A, Opler LA (1987). The positive and negative syndrome scale (PANSS) for schizophrenia. Schizophr Bull.

[23] Lindenmayer JP, Grochowski S, Hyman RB (1995). Five factor model of schizophrenia: replication across samples. Schizophr Res.

[24] Citrome L, Meng X, Hochfeld M (2011). Efficacy of iloperidone in schizophrenia: a PANSS five-factor analysis. Schizophr Res.

[25] Shrout PE, Fleiss JL (1979). Intraclass correlations: uses in assessing rater reliability. Psychol Bull.

[26] Bagby RM, Parker JD, Taylor GJ (1994). The twenty-item Toronto Alexithymia Scale–I Item selection and cross-validation of the factor structure. J Psychosom Res.

[27] Zhu X, Yi J, Yao S, Ryder AG, Taylor GJ, Bagby RM (2007). Cross-cultural validation of a Chinese translation of the 20-item Toronto Alexithymia Scale. Compr Psychiatry.

[28] Yi JY, Yao SQ, Zhu XZ (2003). The Chinese version of the TAS-20: reliability and validity. Chin Mental Health J.

[29] Woods SW (2003). Chlorpromazine equivalent doses for the newer atypical antipsychotics. J Clin Psychiatry.

[30] Sequeira AS, Silva B (2019). A comparison among the prevalence of alexithymia in patients with psychogenic nonepileptic seizures, epilepsy, and the healthy population: a systematic review of the literature. Psychosomatics.

[31] Demirkol ME, Tamam L, Namli Z, Karaytuğ MO, Uğur K (2019). Association of psychache and alexithymia with suicide in patients with schizophrenia. J Nerv Ment Dis.

[32] Rahm C, Liberg B, Reckless G, Ousdal O, Melle I, Andreassen OA (2015). Negative symptoms in schizophrenia show association with amygdala volumes and neural activation during affective processing. Acta Neuropsychiatr.

[33] Moriguchi Y, Komaki G (2013). Neuroimaging studies of alexithymia: physical, affective, and social perspectives. Biopsychosoc Med.

[34] van der Velde J, Servaas MN, Goerlich KS, Bruggeman R, Horton P, Costafreda SG (2013). Neural correlates of alexithymia: a meta-analysis of emotion processing studies. Neurosci Biobehav Rev.

[35] Vrbova K, Prasko J, Holubova M, Slepecky M, Ociskova M (2018). Positive and negative symptoms in schizophrenia and their relation to depression, anxiety, hope, self-stigma and personality traits - a cross-sectional study. Neuro Endocrinol Lett.

[36] Freeman J, Gorst T, Gunn H, Robens S (2020). "A non-person to the rest of the world": experiences of social isolation amongst severely impaired people with multiple sclerosis. Disabil Rehabil.

[37] Li S, Zhang B, Guo Y, Zhang J (2015). The association between alexithymia as assessed by the 20-item Toronto Alexithymia Scale and depression: A meta-analysis. Psychiatry Res.

[38] Leweke F, Leichsenring F, Kruse J, Hermes S (2012). Is alexithymia associated with specific mental disorders?. Psychopathology.

[39] Lenzo V, Barberis N, Cannavò M, Filastro A, Verrastro V, Quattropani MC (2020). The relationship between alexithymia, defense mechanisms, eating disorders, anxiety and depression. Riv Psichiatr.

[40] Son SH, Jo H, Rim HD, Kim JH, Kim HW, Bae GY (2012). A comparative study on alexithymia in depressive, somatoform, anxiety, and psychotic disorders among Koreans. Psychiatry Investig.

[41] Freyberger H (1977). Supportive psychotherapeutic techniques in primary and secondary alexithymia. Psychother Psychosom.

[42] van der Velde J, Swart M, van Rijn S, van der Meer L, Wunderink L, Wiersma D (2015). Cognitive alexithymia is associated with the degree of risk for psychosis. PLoS ONE.

[43] Chen L, Xu L, You W, Zhang X, Ling N (2017). Prevalence and associated factors of alexithymia among adult prisoners in China: a cross-sectional study. BMC Psychiatry.

[44] Chalah MA, Kauv P, Palm U, Lefaucheur JP, Hodel J, Créange A (2020). Deciphering the neural underpinnings of alexithymia in multiple sclerosis. Neurosci Lett.

[45] Lumley MA, Neely LC, Burger AJ (2007). The assessment of alexithymia in medical settings: implications for understanding and treating health problems. J Pers Assess.

